# Molecular epidemiology and carbapenem resistance characteristics of *Acinetobacter baumannii* causing bloodstream infection from 2009 to 2018 in northwest China

**DOI:** 10.3389/fmicb.2022.983963

**Published:** 2022-08-22

**Authors:** Yihai Gu, Wei Zhang, Jine Lei, Lixia Zhang, Xuan Hou, Junqi Tao, Hui Wang, Minghui Deng, Mengrong Zhou, Rui Weng, Jiru Xu

**Affiliations:** ^1^Department of Microbiology and Immunology, School of Basic Medical Sciences, Xi’an Jiaotong University, Xi’an, Shaanxi, China; ^2^Department of Microbiology, 3201 hospital, School of Medicine, Xi’an Jiaotong University, Hanzhong, Shaanxi, China; ^3^Department of Clinical Laboratory, First Affiliated Hospital, Xi’an Jiaotong University, Xi’an, Shaanxi, China; ^4^Department of Clinical Laboratory, Shaanxi Provincial People's Hospital, Xi’an, Shaanxi, China

**Keywords:** *Acinetobacter baumannii*, bloodstream infection, cgMLST, oxacillinase, XerC/D

## Abstract

Bloodstream infection (BSI) caused by *Acinetobacter baumannii* poses a serious threat to health and is correlated with high mortality in patients with hospital-acquired infections, so the molecular epidemiology and antimicrobial resistance characteristics of this pathogen urgently need to be explored. *A. baumannii* isolates from BSI patients were collected in three tertiary hospitals in northwest China from 2009 to 2018. Antimicrobial susceptibility testing was used to determine the MICs of the *A. baumannii* isolates. Whole-genome sequencing based on the Illumina platform was performed for molecular epidemiological analyses and acquired resistance gene screening. The efflux pump phenotype was detected by examining the influence of an efflux pump inhibitor. The expression of efflux pump genes was evaluated by RT-PCR. In total, 47 *A. baumannii* isolates causing BSI were collected and they presented multidrug resistance, including resistance to carbapenems. Clone complex (CC) 92 was the most prevalent with 30 isolates, among which a cluster was observed in the phylogenetic tree based on the core genome multi-locus sequence type, indicating the dissemination of a dominant clone. BSI-related *A. baumannii* isolates normally harbour multiple resistance determinants, of which oxacillinase genes are most common. Except for the intrinsic *bla*_OXA-51_ family, there are some carbapenem-resistant determinants in these *A. baumannii* isolates, including *bla*_OXA-23_, which is encoded within the Tn*2006*, Tn*2008* or Tn*2009* transposon structures and *bla*_OXA-72_. The transfer of *bla*_OXA-72_ was suggested by XerC/D site-specific recombination. The AdeABC efflux pump system contributed to carbapenem resistance in *A. baumannii* isolates, as evidenced by the high expression of some of its encoding genes. Both the clone dissemination and carbapenem resistance mediated by oxacillinase or efflux pumps suggest an effective strategy for hospital infection control.

## Introduction

*Acinetobacter* spp. are opportunistic pathogens observed during clinical infections, among which *Acinetobacter baumannii* is the most clinically significant (Wong et al., 2017). *A. baumannii* normally colonises on the surface of the skin, mucosa, throat and respiratory tract, causing severe infections including bloodstream infections (BSIs), respiratory infections, skin and soft tissue infections, urinary tract infections, meningitis (Harding et al., 2018; Ramirez et al., 2020). The high mortality associated with BSI caused by *A. baumannii* reached up to 45% in a previous study and is a major concern for nosocomial infection control (Zhou et al., 2019; Gu et al., 2021).

Carbapenems have been known as last-resort antibiotics for *A. baumannii* infections, but unfortunately, carbapenem-resistant *A. baumannii* (CRAB) has spread worldwide and the positive rate observed during clinical screening has continued to increase in recent decades, from 1% in 2003 to 58% in 2008, resulting in a major threat to human health and clinical settings (Reddy et al., 2010). The average positive rate for CRAB in China was 53.7% in 2020, as determined using the CARSS surveillance data.^1^

The resistance mechanism of *A. baumannii* against carbapenems is closely related to the hyperproduction of *β*-lactamases, including some AmpC β-lactamases, extended-spectrum β-lactamases (ESBLs) and carbapenemases (Patel and Bonomo, 2013; Stewart et al., 2019). In *A. baumannii*, the most prevalent mechanism responsible for carbapenem resistance is the production of carbapenem-hydrolysing Ambler class D β-lactamases, such as the OXA-23, OXA-24/40, OXA-58, OXA-143 and OXA-235 types (Peleg et al., 2008; Hammoudi and Ayoub, 2020), among which OXA-23-type carbapenemases are most common in CRAB strains spreading worldwide in nosocomial environments (Potron et al., 2015). Carbapenemase-encoding genes are normally located on chromosomes and/or plasmids, and most of them correspond to mobile genetic elements (MGEs), such as insertion sequences (ISs), integrons and transposons. MGEs are responsible for acquiring, transferring or regulating resistance genes within the host. Several studies have described the dissemination of carbapenem resistance genes by MGEs in *A. baumannii*, resulting in difficulties in treating infectious diseases (Roca et al., 2012; Cornejo-Juarez et al., 2020). Sometimes plasmid-borne carbapenemases are flanked by short DNA sequences providing potential recognition sites for the host XerC and XerD site-specific tyrosine recombinases, contributing to the translocation of these resistance genes (Cameranesi et al., 2018). Furthermore, some multidrug efflux systems, such as the resistance-nodulation-cell division (RND) family efflux pump AdeABC, mediate multidrug resistance, including resistance to carbapenems (Coyne et al., 2011).

The goal of this study was to explore the molecular epidemiology and resistance mechanism of *A. baumannii* isolated from BSI patients, providing an efficient therapy choice and reducing the mortality due to BSI caused by *A. baumannii*.

## Materials and methods

### Strains

In total, 47 *A. baumannii* isolates from BSI patients were collected, including four strains collected from Shaanxi Provincial People’s Hospital in 2018, 25 strains from the First Affiliated Hospital of Xi’an Jiaotong University from 2015 to 2018 and 18 strains from the 3201 Hospital from 2009 to 2018. The species of all the isolates were identified by MALDI-TOF (Bruker, Germany) and confirmed by 16S rDNA sequencing and whole-genome sequencing. This study was approved by the Ethics Committees of 3,201 Hospital of Xi’an Jiaotong University School of Medicine (2020005) with a waiver of informed consent because of the retrospective nature of the study.

### Antimicrobial susceptibility testing

The microbroth dilution method was employed to determine the MICs of 47 *A. baumannii* isolates against several antimicrobial agents, including piperacillin/tazobactam, ampicillin/sulbactam, cefepime, ceftazidime, ceftriaxone, cefotaxime, meropenem, imipenem, colistin, gentamicin, amikacin, levofloxacin, ciprofloxacin, tigecycline and cefoperazone/sulbactam. The susceptibility breakpoint was interpreted as recommended by the guidelines of the Clinical and Laboratory Standards Institute (CLSI, 2019), except the breakpoint of tigecycline, which was as recommended by the guidelines of the European Committee on Antimicrobial Susceptibility Testing (EUCAST, 2018). *Escherichia coli* ATCC 25922 and *Pseudomonas aeruginosa* ATCC 27853 were used as quality controls.

### Whole-genome sequencing

Total genomic DNA from the 47 *A. baumannii* isolates was extracted using the QIAamp DNA Minikit (Qiagen, Hilden, Germany) according to the manufacturer’s recommendations. The whole genomes of all isolates were sequenced on the Illumina HiSeq X Ten platform (Illumina, San Diego, CA, United states) *via* the 2 × 150 bp paired-end protocol and were subsequently assembled by using CLC genomic workbench version 8.0, and the draft genome contigs were screened for acquired resistance genes by using ResFinder 2.1 on the CGE server.^2^ The genetic structure surrounding the resistance gene was annotated by BLAST.^3^ The XerC/D-specific recombination site was recognised by PdifFinder.^4^

### Molecular epidemiology based on genome sequence

Multi-locus sequence typing (MLST) analysis was performed by screening the assembly contig sequences of each genome using MLST tool version 2.0 on the CGE website.^5^ The Oxford MLST allele scheme was employed for typing with seven housekeeping genes, including *gltA*, *gyrB*, *gdhB*, *recA*, *cpn60*, *gpi* and *rpoD*. Ridom SeqSphere + software version 4.1.9 (Ridom GmbH Münster, Germany) was used to illustrate the MLST and core genome (cg) MLST relationship. cgMLST analysis was carried out using Paul G. Higgins’ scheme, which employed 2,390 genes in the *A. baumannii* genome as core genes. The paired isolates that differed by less than ten core genes were deemed closely related. A phylogenetic tree based on cgMLST was generated by using the minimum spanning tree (AST) algorithm.

### Detection of the efflux pump phenotype

Overexpression of the efflux pump phenotype is usually observed when there is a significant increase in carbapenem susceptibility when an isolate is incubated with a carbapenem and the appropriate efflux pump inhibitor (Mmatli et al., 2020). Carbonyl cyanide m-chlorophenylhydrazine (CCCP), phenylalanine-arginine *β*-naphthylamide (PAβN) and 1-(1-naphthylmethyl)-piperazine (NMP) were used as inhibitors to assess the potential decrease of MIC of carbapenem in BSI-related *A. baumannii* isolates.

The expression of the genes *adeA* and *adeB*, which belong to the multidrug efflux pump AdeABC, was assessed in efflux phenotype-positive isolates by RT-PCR. RNA from the isolates was extracted by using the PureLink RNA Mini Kit (Invitrogen, Carlsbad, CA, United States) in the exponential growth period of bacterial cells and was subsequently reverse transcribed to cDNA by the PrimeScript^™^ RT Reagent Kit (Takara, Kyoto, Japan). The gene expression level was evaluated by using TB Green^™^ Premix Ex Taq^™^ (Takara, Kyoto, Japan) in a LightCycler 480 system (Roche, Rotkreuz, Switzerland) with triplicate samples for each isolate, and three replicates were performed independently using the 2^–ΔΔCT^ method with previously reported primers. Genes for which the fold change in expression was greater than 2 were considered to be differentially expressed. The housekeeping gene *rpoB* was used as the internal reference, and the strain ATCC17978 was used as a reference control.

## Results

### Antimicrobial susceptibility testing

All 47 *A. baumannii* isolates causing BSI presented high-level resistance against most antimicrobial agents, including β-lactams/β-lactamase inhibitors, third/fourth generation cephalosporins, quinolones, tetracyclines, aminoglycosides and even carbapenem, the last-resort antibiotic for severe infections caused by Gram-negative bacteria. The resistance rates for piperacillin/tazobactam, ceftriaxone, cefepime, meropenem, imipenem, ciprofloxacin, tigecycline and gentamicin were greater than 70%. Colistin showed the highest susceptibility rate (97.9%) among all the tested antimicrobial agents, followed by ceftazidime (Table 1). During the decade of strain collection, we further selected two periods that possessed relatively more isolates to observe the trend of carbapenem resistance. The early period had 13 isolates and was from 2013 to 2015, and the later period had 33 isolates from 2016 to 2018. We found that the carbapenem resistance rate of BSI-related *A. baumannii* increased from 61.5% during the early stage to 72.7% during the later stage.

**Table 1:** Antimicrobial susceptibility testing results of 47 *Acinetobacter baumannii* isolates causing bloodstream infection (BSI).

Antimicrobial agents	Resistance rate (%)	Intermediate rate (%)	Susceptible rate (%)
Ampicillin/sulbactam	55.3	6.4	38.3
Piperacillin/tazobactam	72.3	6.4	21.3
Cefoperazone/sulbactam	61.7	6.4	31.9
Ceftazidime	27.7	0	72.3
Ceftriaxone	72.3	19.1	8.5
Cefotaxime	63.8	6.4	29.8
Cefepime	72.3	6.4	21.3
Meropenem	72.3	0	27.7
Imipenem	72.3	0	27.7
Levofloxacin	66.0	4.3	29.8
Ciprofloxacin	70.2	0	29.8
Tigecycline	72.3	8.5	19.1
Colistin	2.1	0	97.9
Gentamicin	70.2	0	29.8
Amikacin	61.7	2.1	36.2

### Molecular epidemiology

**Figure 1:**
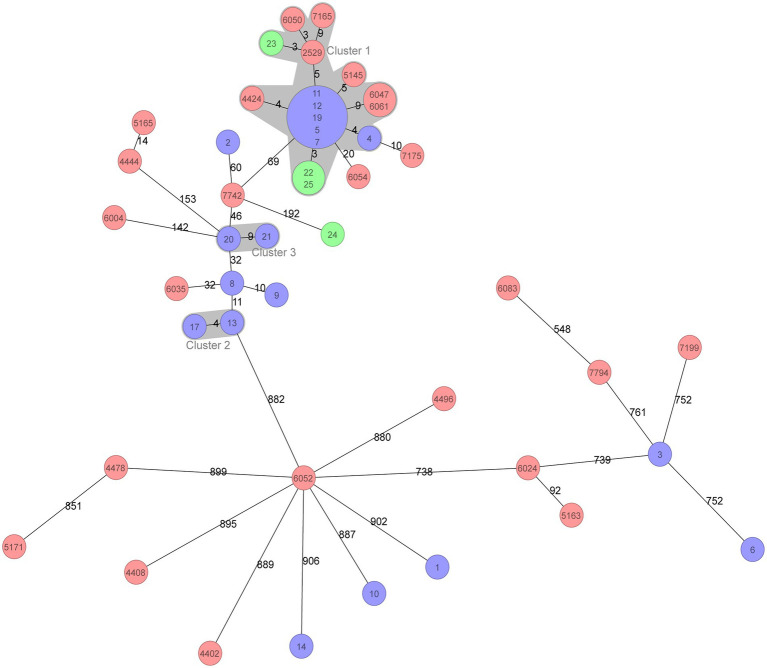
Minimum spanning tree of 47 BSI *Acinetobacter baumannii* isolates based on cgMLST. cgMLST profiles are represented by circles, and the isolate name is marked on the circle. The size of the circle is proportional to the number of isolates with an identical cgMLST profile. The different colours in the circle represent the three hospitals from which the isolates were collected. The number on the line connecting the cgMLST circles is the number of core genes that differ between the isolates within the circles. The grey zone surrounding a group of circles represents the closely related isolates that differed by less than ten core genes.

All 47 *A. baumannii* isolates were distributed into 22 STs based on the Oxford MLST scheme, among which ST195 was 0the most dominant, with 17 isolates (36.2%), followed by ST208 with 8 isolates (17.0%). Except for two ST218 and two ST1818 isolates, each of the remaining isolates belonged to a single ST. Clone complex (CC) 92 was the most prevalent with 30 isolates (63.8%) and encompassed six STs, including ST195 and ST208. Strikingly, all of the CC92 isolates (30/30, 100%) exhibited resistance against carbapenems, whereas 4 of 17 (23.5%) of the other ST isolates were carbapenem-resistant. cgMLST analysis was subsequently performed to assess the phylogenetic relationship of these BSI-related *A. baumannii* isolates with higher resolution based on genome sequences. There was a large relevant cluster (cluster 1 in Figure 1) observed in the phylogenetic tree, the isolates in which originated from all three different hospitals and belonged to ST195, indicating that a dominant clone was disseminated among the hospitals.

### Resistome analysis

**Figure 2:**
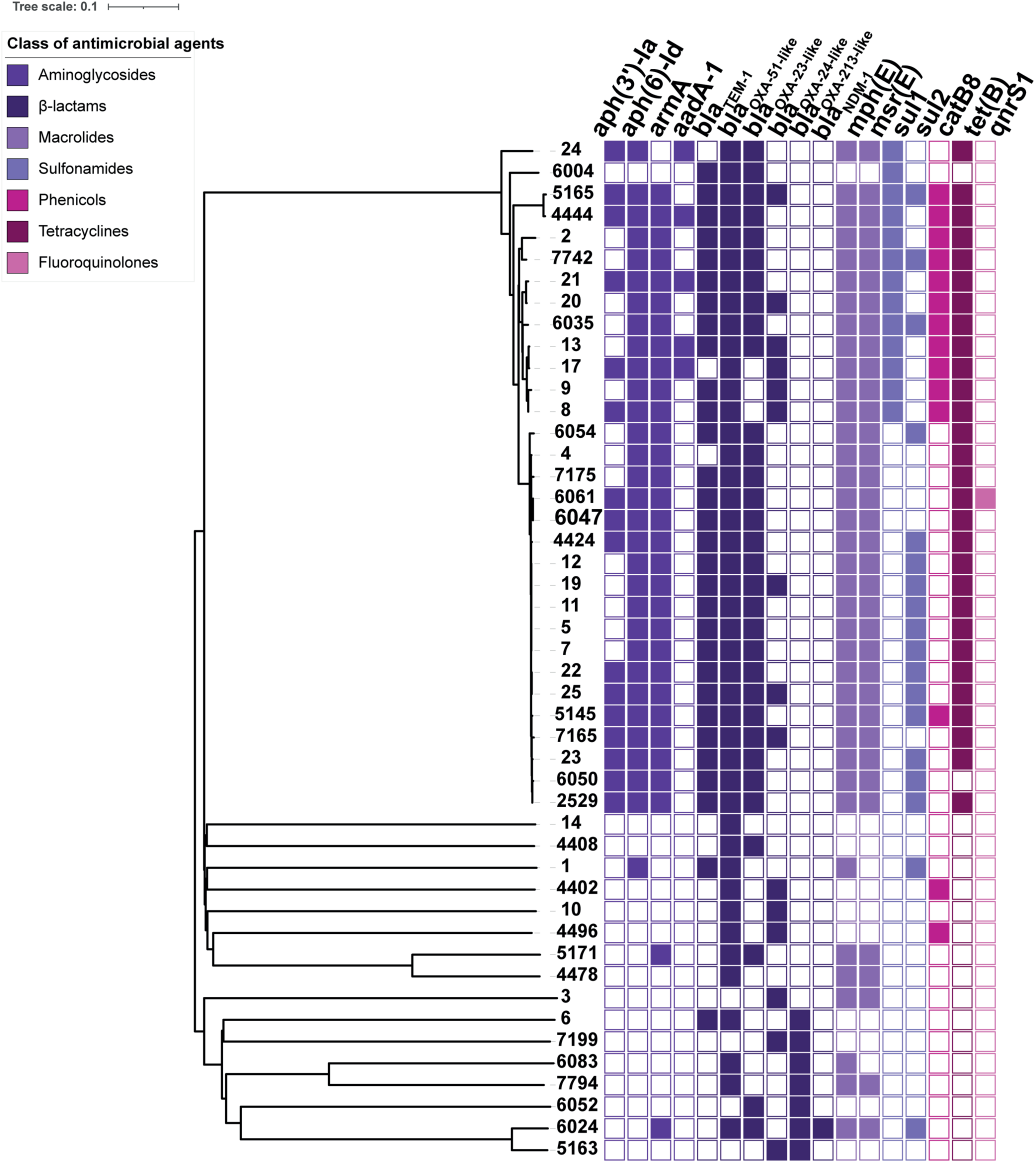
Phylogenetic relationship based on the core genome among 47 BSI *A. baumannii* isolates and the heatmap of resistance determinants. In the heatmap, colour-filled rectangles indicate the positive resistance determinants, and colour-empty rectangles indicate the negative resistance determinants. Different colours represent the classes of antimicrobial agents to which the isolate confers resistance.

Most of these BSI-related *A. baumannii* isolates (46/47) harboured intrinsic *bla*_OXA-51-like_ or *bla*_OXA-213-like_ genes, including *bla*_OXA-51_, *bla*_OXA-66_, *bla*_OXA-80_, *bla*_OXA-88_, *bla*_OXA-106_, *bla*_OXA-111_, *bla*_OXA-120_, *bla*_OXA-132_, *bla*_OXA-430_, *bla*_OXA-273_, *bla*_OXA-421_, *bla*_OXA-500_, *bla*_OXA-526_ and *bla*_OXA-533_, by which the oxacillinase expression did not mediate carbapenem resistance. Several other OXA-type genes, such as *bla*_OXA-23_ and *bla*_OXA-72_, were mainly responsible for carbapenem resistance. The *bla*_OXA-23_ carbapenemase gene was most common and was present in 32 isolates, one of which co-harboured another metal-*β*-lactamase, *bla*_NDM-1_. There were also 15 *bla*_OXA-72_-positive isolates. Moreover, 31/47 isolates harboured the 16S rRNA methylase gene *armA*, which was responsible for high-level resistance against aminoglycosides. Aminoglycoside-modifying enzymes that commonly mediate low-or medium-level resistance to aminoglycosides, such as *aph(3′)-Ia*, *aph(6)-Id* and *aadA-1*, were detected in 32 *A. baumannii* isolates. Among other antimicrobial resistance genes, we also screened the macrolide resistance genes *mph(E)* and *msr(E)*, sulphonamide resistance genes *sul1* and *sul2*, tigecycline resistance gene *tet(B)*, phenicol resistance gene *catB8* and fluoroquinolone resistance gene *qnrS1* (Figure 2).

### Genetic structure of carbapenemase genes

The genetic structure of the acquired carbapenemase genes was further analysed. The spread of the carbapenemase gene *bla*_OXA-23_ is normally mediated by several known transposons, such as Tn*2006*, Tn*2007*, Tn*2008* and Tn*2009*, among which the distinct genetic structure was found to be distributed on IS*Aba1*/IS*Aba4* or the dual-copy insertion of IS*Aba1*. The known transposon structures were screened in all *bla*_OXA-23_-positive isolates, and the comparison results indicated that 21/32 (65.6%) were connected to Tn*2006*, 8/32 (25.0%) were Tn*2009* and the remaining 3/32 (9.4%) were connected to Tn*2008*. Conjugation assays showed that the *bla*_OXA-23_ gene was located on the plasmid in at least 9/32 *bla*_OXA-23_-positive isolates, which successfully acquired the *bla*_OXA-23_-harbouring plasmid.

**Figure 3:**
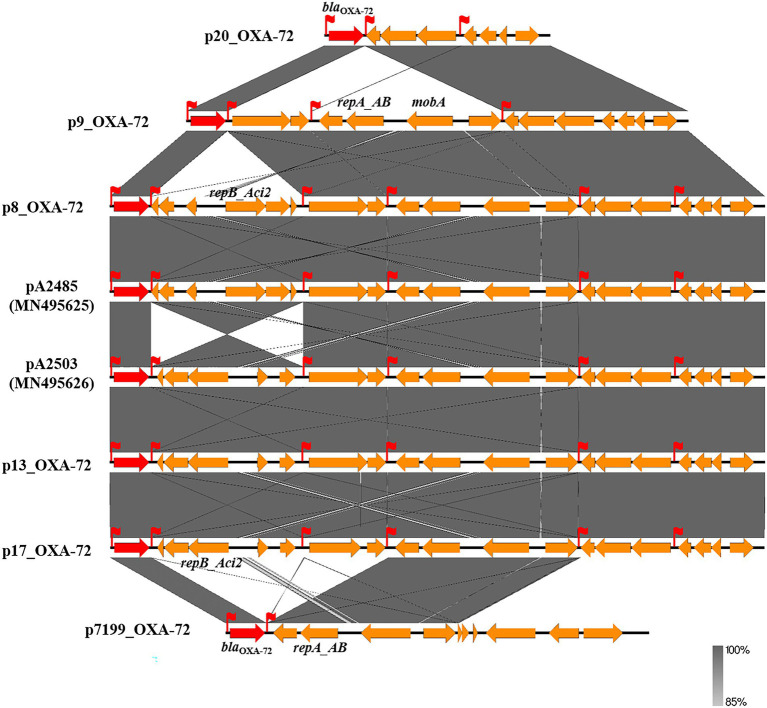
Sketch and comparison of the genetic structure of *bla*_OXA-72_-harbouring large contigs. Orange rectangular arrows on the line represent the open reading frames (ORFs) encoded by the contig sequence, and the red arrows represent the *bla*_OXA_-gene. The dark grey shadows indicate the 72 identical sequence segment between two contigs. The red flags represent the XerC/XerD-like combination site.

The *bla*_OXA-40_ variant *bla*_OXA-72_ belonged to the *bla*_OXA-24_ cluster and was identified in 15/47 (31.9%) *A. baumannii* isolates. The *bla*_OXA-72_-containing contig sequences were extracted from the genome data, among which six contigs from isolates 8, 9, 13, 17, 20 and 7,199 were greater than 2 kbp in size and were screened for homology against the GenBank database. The results showed that these six *bla*_OXA-72_-containing contigs were approximately 6–15 kb in size and 100% identical or partly similar to plasmids pA2503 (MN495626) and pA2485 (MN495625), which are both 15,405 bp in size. Notably, no mobile element, such as a transposon or IS, was found surrounding the *bla*_OXA-72_ gene (Figure 3). However, interestingly, the genetic structure comparison illustrated that several insertions, deletions or inversions occurred among these plasmid segments, and on the border of the fragment, we found some pairs of XerC/XerD-like sites, which could provide active pairs for site-specific recombination mediating horizontal gene transfer. For example, a pair of XerC/XerD-like sites were found at the border of a 5 kbp inversion between isolate 8 and isolate 13 (or 17). Similarly, XerC/XerD-like sites also emerged at the border of in/del segments between isolates 20 and 9, isolates 9 and 8, isolates 7,199 and 17, etc. Crucially, the *bla*_OXA-72_ gene was observed as a segment flanked closely by XerC/XerD-like sites, suggesting that XerC/XerD-like site-mediated recombination may be responsible for mobilisation of the *bla*_OXA-72_ gene in the BSI-related *A. baumannii* isolates in our study (Figure 3).

### Detection of the efflux pump phenotype

**Figure 4:**
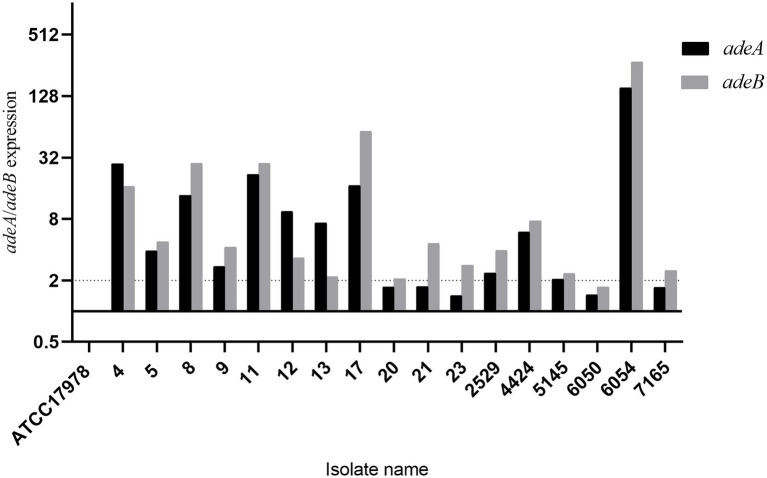
Expression of the AdeABC efflux pump genes *adeA* and *adeB*. ATCC17978 was used as a baseline.

In total, 25/47 (53.2%) isolates presented an efflux pump phenotype that decreased MIC of meropenem or imipenem by 4-fold or more. Most (23/25, 92.0%) of the efflux pump positive isolates were identified under the CCCP inhibitor, and six isolates tested positive under PAβN. The inhibitor NMP did not induce an efflux pump phenotype in any of the isolates. Eighteen isolates that showed a more than 64-fold decrease in the MIC of meropenem were selected for subsequent evaluation of the expression of the genes *adeA* and *adeB* in the multidrug efflux pump system AdeABC. The results showed that 17/18 (94.4%) isolates presented higher expression of *adeA* or *adeB* than the reference strain ATCC 17978, among them 16 isolates presented a more than 2-fold (significant) increase in expression of at least one pump gene (Figure 4). The last isolate exhibited no expression of either the *adeA* or *adeB* gene due to deletion of the AdeABC efflux pump, which was confirmed by PCR on chromosome DNA.

## Discussion

BSI caused by *A. baumannii* is associated with high patient mortality and is one of the most urgent threats to public health. In this study, 47 *A. baumannii* isolates causing BSI were collected in three tertiary hospitals over a decade. Antimicrobial susceptibility testing and whole-genome sequencing were performed to assess the molecular epidemiology and antimicrobial resistance characteristics of the isolates. Multidrug resistance, especially resistance against carbapenem, was observed in these BSI-related *A. baumannii* isolates, leaving limited choice for therapy. Colistin, a cationic polypeptide belonging to the polymyxin family, has been introduced into clinical practice as an important therapeutic option for carbapenem-resistant Gram-negative bacterial infections (Falagas et al., 2011). A previous study indicated that colistin monotherapy was associated with a better outcome than colistin-meropenem combination therapy (Dickstein et al., 2019). However, using colistin as a therapy choice still depends on PK/PD and underlying complications due to its potential renal toxicity.

Molecular epidemiology based on genomic data facilitated the investigation of the dissemination and phylogenetic relationship of BSI-related *A. baumannii* isolates with higher distinguishability. CC92 clone dissemination was observed in this study accounting for more than half of the BSI-related *A. baumannii* isolates. CC92 is the most common clone complex in *A. baumannii* from China (Karah et al., 2012). More importantly, all isolates belonging to the CC92 clone were carbapenem-resistant, which was one of the crucial reasons they survived under high antibiotic pressure and spread widely, consequently inducing BSI. cgMLST was used to further discover the diffusion of a dominant clone among three different hospitals in northwest China, as evidenced by the presence of a cluster comprising genetically indistinguishable isolates.

The BSI-related *A. baumannii* isolates harboured multiple resistance determinants, among which the oxacillinase genes were the most common. The OXA-type enzymes in *A. baumannii* are normally divided into four clusters based on their genetic similarities, namely, the OXA-51, OXA-23, OXA-24 and OXA-58 clusters (Peleg et al., 2008). The enzymes of the OXA-51 cluster are naturally occurring enzymes in *A. baumannii* given their chromosomal location and minimal effect on carbapenem susceptibility. More than ten variants of *bla*_OXA-51_ or *bla*_OXA-213_ were observed in 46 of the BSI-related *A. baumannii* isolates, indicating the diversity of the intrinsic oxacillinase gene cluster.

In the *Acinetobacter* genus, the acquired carbapenem hydrolysing oxacillinases contribute to carbapenem resistance. OXA-23 was the first identified carbapenemase, and its role in carbapenem-hydrolysis appeared to be elevated in the presence of the upstream IS*Aba1* element. In our study, the *bla*_OXA-23_-positive isolates were associated with several known transposons, Tn*2006*, Tn*2008* and Tn*2009*, and all of them encompassed the IS*Aba1* element that possibly mediated their high-level carbapenem resistance (Chen et al., 2017). Yang’s study reported 58 *A. baumannii* strains carrying *bla*_OXA-23_ gene in China, 47 isolates (47/58, 81.0%) were associated with Tn*2009* and 8 isolates (8/58,13.8%) were associated with Tn*2006* (Yang et al., 2019). Another study reported by Cerezalesa showed that 51 carbapenem-resistant *A. baumannii* strains carried the *bla*_OXA-23_ gene in transposon Tn*2008* (Cerezales et al., 2019). In our study, the transposons that harboured the *bla*_OXA-23_ gene in BSI-related *A. baumannii* in Shaanxi province presented diverse, and the Tn*2006* was the most common (65.6%) in our report, suggesting a distinction from the results of previous studies.

The *bla*_OXA-40_ variant *bla*_OXA-72_ belongs to the *bla*_OXA-24_ cluster, and its presence has been reported in several previous studies on CRAB (Kuo et al., 2013; Dortet et al., 2016; Chen et al., 2018). Analysis of the *bla*_OXA-72_ genetic environment revealed a potential transfer mechanism corresponding to a recombination site. In *A. baumannii* and most bacteria, dimers are resolved to monomers by site-specific recombination, which is a process that is performed by two chromosomally encoded tyrosine recombinases (XerC and XerD). Several studies have reported that plasmid-borne *bla*_OXA_-containing structures are bordered by short sequences exhibiting homology with the 28-nucleotide dif motif located at the bacterial chromosome replication terminus and are recognised by XerC/D site-specific recombinases, leading to the hypothesis that their mobilisation could be mediated by site-specific recombination (Merino et al., 2010). In our study, all *bla*_OXA-72_-containing contigs were found to have a pair of 28-nucleotide XerC/XerD-like sites that closely flanked the *bla*_OXA-72_ gene, indicating that recombination through the Xer system likely occurs to mediate transfer of the carbapenem-resistance gene to the nosocomial environment.

Efflux pumps often play a crucial role in multidrug resistance in *A. baumannii*, including by mediating a possibly significant increase in carbapenem susceptibility (Abdi et al., 2020). In this study, the carbapenem susceptibility in more than half of the BSI-related *A. baumannii* isolates was influenced by efflux pump inhibitors. The inhibitor CCCP seemed more efficient for the majority of isolates with an efflux pump phenotype, and in contrast, the inhibitor NMP showed no impact. The AdeABC efflux pump is a member of the RND family and can pump out multiple antibiotics, and overexpression of the AdeABC efflux pump may confer high-level resistance to carbapenems (Zhu et al., 2013). The majority of efflux pump phenotype-positive isolates showed higher expression of AdeABC efflux pump genes, except one isolate, which lacked these genes. The presence of other functional efflux pumps in that isolate could potentially explain the efflux pump-positive phenotype.

In conclusion, this study of molecular epidemiology and antimicrobial resistance characteristics revealed that *A. baumannii* isolates causing BSI presented clone dissemination and multidrug resistance. The multidrug-resistant clone CC92 had spread among distinct hospitals in northwest China over decade-long period of our study. The BSI-related *A. baumannii* isolates consistently exhibited resistance against carbapenems, which was attributed to the wide distribution of oxacillinases OXA-23 and OXA-72. In addition to the carbapenemases produced, the efflux pump harboured by the *A. baumannii* isolates also plays an important role, and the efflux pump genes were suggested to exhibit significantly increased expression. BSI caused by *A. baumannii* isolates poses a serious threat to health and is correlated with high mortality in patients with hospital-acquired infections. Additional strategies for nosocomial infection control urgently needed to prevent these multidrug-resistant *A. baumannii* clones from becoming endemic.

## Data availability statement

The datasets presented in this study can be found in online repositories. The names of the repository/repositories and accession number(s) can be found at: https://www.ncbi.nlm.nih.gov/, PRJNA844406.

## Author contributions

JX conceived and designed this study. YG, JL, and LZ collected strains of bacteria. YG, JL, and JT collected the isolates and clinical data. WZ and HX performed the antimicrobial susceptibility testing. WZ, HW, JT, MD, and MZ carried out whole genome sequencing and analysis. YG and RW structured the variables and performed the statistical analyses. YG wrote the manuscript. All authors contributed to the article and approved the submitted version.

## Funding

This work was supported by the Key R&D project of Shaanxi Provincial Department of Science and Technology (no. 2019SF-220).

## Conflict of interest

The authors declare that the research was conducted in the absence of any commercial or financial relationships that could be construed as a potential conflict of interest.

## Publisher’s note

All claims expressed in this article are solely those of the authors and do not necessarily represent those of their affiliated organizations, or those of the publisher, the editors and the reviewers. Any product that may be evaluated in this article, or claim that may be made by its manufacturer, is not guaranteed or endorsed by the publisher.

## Footnotes

^1^ http://www.carss.cn/

^2^ https://cge.cbs.dtu.dk/

^3^ https://blast.ncbi.nlm.nih.gov/

^4^ http://pdif.dmicrobe.cn/pdif/analysis/

^5^ https://cge.cbs.dtu.dk/services/MLST/
